# A decade of faculty development for health professions educators: lessons learned from the Macy Faculty Scholars Program

**DOI:** 10.1186/s12909-023-04155-x

**Published:** 2023-03-27

**Authors:** Mary Haas, Justin Triemstra, Marty Tam, Katie Neuendorf, Katherine Reckelhoff, Rachel Gottlieb-Smith, Ryan Pedigo, Suzy McTaggart, John Vasquez, Edward M. Hundert, Bobbie Berkowitz, Holly J. Humphrey, Larry D. Gruppen

**Affiliations:** 1 Department of Emergency Medicine, University of Michigan Medical School, Ann Arbor, MI USA; 2 Department of Pediatrics and Human Development, Corewell Health, Helen DeVos Children’s Hospital, Michigan State University College of Human Medicine, Grand Rapids, MI USA; 3 Division of Cardiovascular Medicine, University of Michigan, Ann Arbor, MI USA; 4 Department of Palliative and Supportive Care, Clevel and Clinic Lerner College of Medicine of Case Western Reserve University, Cleveland, OH USA; 5 College of Chiropractic, Cleveland University, Kansas City, Overland Park, KS USA; 6 School of Medical & Applied Sciences, Central Queensland University, Brisbane City, QLD Australia; 7 Department of Pediatrics, University of Michigan Medical School, Ann Arbor, MI USA; 8 Department of Emergency Medicine, Harbor-UCLA Medical Center, Emergency Medicine, David Geffen School of Medicine at UCLA, Los Angeles, LA USA; 9 Office of Medical Student Education, University of Michigan Medical School, Ann Arbor, MI USA; 10 Arapahoe Community College, Littleton, CO USA; 11 Medical Education, Harvard University, Harvard Medical School, Boston, MA USA; 12 Columbia University School of Nursing and University of Washington School of Nursing, Seattle, WA USA; 13 Josiah Macy, Jr. Foundation, New York, NY USA; 14 The University of Chicago, Chicago, IL USA; 15 Department of Learning Health Sciences, University of Michigan Medical School, Ann Arbor, MI USA

**Keywords:** Faculty development, Program evaluation, Health professions education

## Abstract

Faculty development (FD) programs are critical for providing the knowledge and skills necessary to drive positive change in health professions education, but they take many forms to attain the program goals. The Macy Faculty Scholars Program (MFSP), created by the Josiah Macy Jr. Foundation (JMJF) in 2010, intends to develop participants as leaders, scholars, teachers, and mentors. After a decade of implementation, an external review committee conducted a program evaluation to determine how well the program met its intended goals and defined options for ongoing improvement.

The committee selected Stufflebeam’s CIPP (context, input, process, products) framework to guide the program evaluation. Context and input components were derived from the MFSP description and demographic data, respectively. Process and product components were obtained through a mixed-methods approach, utilizing both quantitative and qualitative data obtained from participant survey responses, and curriculum vitae (CV).

The evaluation found participants responded favorably to the program and demonstrated an overall increase in academic productivity, most pronounced during the two years of the program. Mentorship, community of practice, and protected time were cited as major strengths. Areas for improvement included: enhancing the diversity of program participants, program leaders and mentors across multiple sociodemographic domains; leveraging technology to strengthen the MFSP community of practice; and improving flexibility of the program.

The program evaluation results provide evidence supporting ongoing investment in faculty educators and summarizes key strengths and areas for improvement to inform future FD efforts for both the MFSP and other FD programs.

**Supplementary Information:**

The online version contains supplementary material available at 10.1186/s12909-023-04155-x.

## Introduction

### Background

Robust faculty development (FD) programs in the health professions are necessary to provide faculty with the knowledge and skills required to prepare learners for an ever-evolving healthcare landscape. Although FD initially emerged as a form of training to develop teaching skills, it has since expanded to include activities that improve knowledge and skills in other domains such as research, administration and clinical skills [1–3]. In addition to benefiting learners, FD programs can augment the careers of health professions educators through improved self-efficacy and sense of belonging within the broader education community [4]. On an institutional level, FD programs can define and strengthen a medical center’s academic profile [5]. Similarly, national FD programs may benefit from the reputation of the sponsoring institution and its particular goals and mission.

Despite the importance of FD programs, there exist several barriers to participation, including: a lack of protected time for attendees, competing responsibilities, a perceived lack of recognition and financial reward for teaching, a perceived lack of direction from and connection to the institution, and logistical factors [6]. Lack of protected time specifically remains the most commonly reported barrier to scholarship, which is a key output of many FD programs. This constraint is particularly true for faculty managing competing administrative and leadership activities, additional key outputs of FD programs [7, 8]. Previous FD literature has identified dedicated time and financial resources as primary drivers for FD program participants’ ability to actualize benefits of participation, by creating capacity for learning, reflection, scholarship and development of leadership skills [8–10]. The importance of FD programs has resulted in the growth in the number and size of such programs, with corresponding financial commitments. Supporting evidence for ongoing investment in FD programs, as well as good stewardship of this investment of money, time, and people, requires evaluation of the intended and unintended outcomes of such programs.

One such flagship program is the Macy Faculty Scholars Program (MFSP). The MFSP is a FD program created by the Josiah Macy Jr. Foundation (JMJF) in 2010 with the goal of developing health professions educators as leaders, scholars, teachers and mentors [11]. The MFSP selects mid-career faculty who have already demonstrated promise, and subsequently provides them with a structured curriculum, protected time, mentorship, a national network, and a community of practice (CoP). The vision of the program was for Scholars to become “the drivers for change in health professions education, toward the goal of creating an educational system that better meets the needs of the public.” Specifically, the JMJF noted interest in projects that: advance equity, diversity and belonging; enhance collaboration among health professionals, educators, and learners; and successfully navigate and address ethical dilemmas that arise when principles of the health professions conflict with barriers imposed by the health delivery system.

The program selects 5 Scholars per year from institutional nominations through a rigorous, multi-level selection process. Accepted Scholars receive base salary support plus fringe benefits to protect at least 50% of the Scholar’s time for two years. Scholars produce academic output, engage in the national MFSP educational network, and participate in the Harvard Macy Institute Program for Educators in Health Professions [12]. Applicants are selected based on their application, project proposal, institutional support and are mentored throughout the program by a national group of mentors.

To date, the JMJF had not yet engaged in a significant, formal, ongoing evaluation of the MFSP, as the priorities thus far had included building the program, recruiting the mentors, developing selection criteria, partnering with the Harvard Macy Institute for the curriculum, and refining various policies. More generally, rigorous evaluations utilizing both qualitative and quantitative methods of robust national FD programs often remain neglected or considered as an afterthought in the literature, highlighting the need to publish program evaluations that are completed in an academically rigorous manner for transparency of program outcomes [13–15]. Therefore, at the decade mark, the JMJF elected to commission an external review of the MFSP to evaluate components of the program, namely: curriculum, mentorship models, selection, and program and Scholar outcomes. Consequently, the authors conducted a robust program evaluation of the MFSP which is detailed in this paper. Subsequently, this paper can serve as an example of a academically rigorous program evaluation utilizing both quantitative and qualitative methodology and as additional data for other health profession educational leaders seeking to justify, build, or evaluate other FD programs to demonstrate the value of investment in health professions educators [16–18].

## Methods

### Evaluation framework

The evaluation team elected to apply Stufflebeam’s CIPP evaluation framework, which contains four major levels of evaluation: *Context* (program objectives and the basis for those objectives), *Input* (assessment of the educational strategies employed to meet the objectives), *Process* (actual implementation and how it compares to planned activities) and *Product* (resulting outcomes and if they met the needs of the target population).[19, 20]. The CIPP model avoids an overly simplistic approach by incorporating systems theory, which postulates that the whole equals more than the sum of its parts and acknowledges the interrelations among individual components. CIPP also incorporates complexity theory, which accounts for the relationship of elements to program participants and relationship of program participants with one another [19, 21–23]. Although this evaluation occurred at the 10-year-mark, the Macy Foundation leadership intended to use data gleaned both summatively to assess its return on investment, as well as formatively to inform future iterations of the program.

We obtained the context and input components of the evaluation from the MFSP program description and Scholar demographic data, respectively. We applied a mixed-methods approach to evaluate the process and product, utilizing both quantitative and qualitative data obtained from program statistics, survey responses and curriculum vitae (CV). Informed consent was obtained from all subjects and the study was reviewed by the University of Michigan Institutional Review Board, HUM00175014, for ethical approval and ruled to be exempt from ongoing review. All methods and experimental protocols were approved and carried out in accordance with relevant guidelines and regulations.

### Curriculum Vitae (CV) analysis

MFSP Scholars with a minimum of one year of post-program activity submitted current CVs for analysis of types and number of academic products. We aggregated and de-identified the CVs. We used three time periods to classify academic output: time period 1 (pre-MFSP) represents years before the program (equals number of years in time period 3); time period 2 (intra-MFSP) represents the two years during the program; and time period 3 (post-MFSP) represents years after the program up until the end of 2019, ranging from 1 year, for those who entered in 2016, up to 6 years for those who entered in 2011, with a mean of 3.5 years. Nine of the authors (RG, MH, LG, PM, KN, RP, KR, MT, JT) participated in data extraction and performed exploratory data analyses including a review of data distributions by histogram and Q-Q plots, as well as descriptive statistics such as mean, median, variance, skewness, and kurtosis, and outlier analyses using interquartile range and Cook’s distance (Additional file 1: Appendix 1 for CV data collection form).

### Survey

The evaluation team developed survey questions (see Additional file 1: Appendix 2 for complete survey) through an iterative process of expert input and literature review, with content focused on strengths and areas of improvement of the MFSP program. The evaluation team piloted the instrument, refined the questions accordingly, and generated a Qualtrics survey requiring roughly 5- to 10-min to complete. A set of representative MFSP Scholars did not pilot the survey due to the small size of the cohort and potential for generating bias from seeing the questions twice. The MFSP Scholars received the survey via email. Responses were confidential and de-identified in the analysis.

### Quantitative analysis

For the quantitative analysis, we used demographic data, data extracted from the CV analysis, and survey items with Likert-type scale questions. We expressed number of academic outputs from the CV analysis as rates (number per year for a given time period). We utilized descriptive statistics and analysis of variance (ANOVA) to examine the results. We log-transformed numerical data to meet assumptions of normality for the statistical tests prior to comparison between groups. We used a one-way, within subjects ANOVA to test within-subject differences in outcomes during time periods 1, 2, and 3 for statistical significance. We used partial eta-square as effect size measures. Post-hoc analyses assessed the effect of protected time on these outcomes by an analysis of covariance (ANCOVA) using protected time as a covariate. All statistical tests were two-sided with a 0.05 significance level. We performed analyses with IBM SPSS version 26.0 (Armonk, NY).

### Qualitative analysis

Following the Standards for Reporting Qualitative Research, we utilized a constructivist approach to engage in a qualitative analysis of open-ended survey responses to further understand the participants' experience with the MFSP program [24]. With regard to the positionality and reflexivity of the coding group, a gender and experientially diverse group of health professions educators comprised the coding team. Four authors (JDT, RP, KN, KR) reviewed and analyzed transcripts under the direction of a qualitative research expert (JV). All coding authors used a group format to create and discuss code agreement for three questions. Two teams split the remaining questions (authors KN/RP and authors JDT/KR) and each team coded half the questions independently. The independent teams then convened to discuss all codes and determine appropriate themes.

## Results

### Quantitative analysis (demographic, CV, survey)

Table 1 details baseline demographic data of MFSP applicants and selected Scholars as a component of the *inputs* in the CIPP model for the MFSP program.

**Table 1:** MFSP Applicant and Scholar Demographics (2011–2019)

	**Total**	**Percent of total**
**Applicants**	747	n/a
School		
School of Medicine	416	55.7%
School of Nursing	328	44.0%
Both	2	0.3%
Gender		
Female	595	79.5%
Male	153	20.5%
Under-Represented in Medicine (URM)^a^	50^b^	12.6%^b^
**Scholars**	46	
Gender		
Female	34	73.9%
Male	12	26.1%
URM^a^	11	23.9%
Geographic location^c^		
W	7	15.6%
MW	9	20.0%
NE	17	37.8%
S	12	26.7%
Doctorate degree		
PhD (nursing)	14	30.4%
MD	22	47.8%
PhD (other^d^)	5	10.9%
MD/PhD	3	6.5%
PharmD	1	2.2%
MSN/PhD	1	2.2%
Academic Rank		
Assistant professor	16	34.8%
Associate professor	28	60.9%
Professor	2	4.3%

^a^Black/African American, Latinx, Native American, Pacific Islander

^b^Data only available for years 2015–2019

^c^Geographic region categorization based on US Census Bureau

^d^Most common PhD fields of study were education, sociology and psychology

Table 2 summarizes the *products* of the CIPP model*,* or rates of academic outputs per year for each category of the CV analysis over each time period and results of the statistical analysis to determine between-group differences and effect size. A total of 31 CVs met criteria for inclusion in the CV analysis.

**Table 2:** Rate of accomplishments per year by MFSP^a^ participants by time period^b^

Accomplishment^c^	Scholars	Time Period 1, mean (SD)	Time Period 2, mean (SD)	Time Period 3, mean (SD)	*P*-value	Effect size^d^
Honors						
Total	31	1.08 (1.01)	0.89 (0.95)	0.69 (0.53)	0.382	0.021
Education		0.53 (0.72)	0.50 (0.62)	0.41 (0.46)	0.838	0.004
Grants						
Total	31	0.98 (0.69)	0.76 (0.78)	0.65 (0.80)	0.102	0.049
Education		0.70 (0.62)	0.66 (0.78)	0.35 (0.56)	0.056	0.062
Grants as PI						
Total	31	0.60 (0.61)	0.39 (0.51)	0.36 (0.41)	0.143	0.042
Education		0.43 (0.48)	0.21 (0.34)	0.17 (0.28)	**0.020**	0.083
Grant Dollars^e^						
Total	26	$530,486.33 ($804,007.40)	$425,872.83 ($722,805.71)	$465,532.97 ($654,287.27)	**0.029**	0.090
Education		$379,494.93 ($801,996.52)	$259,150.29 ($522,980.14)	$199,151.57 ($334,481.65)	0.228	0.039
Grant Dollars as PI						
Total	26	$229,439.50 ($707,683.87)	$89,594.87 ($339,531.76)	$145,771.15 ($320,325.69)	0.115	0.056
Education		$180,148.90 ($690,156.02)	$3,343.79 ($9,328.59)	$59,060.58 ($200,523.64)	0.173	0.046
Publications						
Total	31	2.21 (2.11)	3.55 (2.87)	3.21 (2.48)	0.052	0.064
Education		1.31 (1.59)	2.10 (2.25)	2.05 (2.00)	0.125	0.045
First Author						
Publications	31	0.98 (0.95)	1.32 (1.11)	1.21 (0.97)	0.339	0.024
Total Education		0.58 (0.55)	0.92 (0.86)	0.74 (0.77)	0.250	0.030
Last Author						
Publications	31	0.47 (0.69)	0.68 (0.79)	0.71 (0.83)	0.366	0.022
Total Education		0.29 (0.60)	0.37 (0.68)	0.52 (0.60)	0.201	0.035
All Presentations						
Total	31	4.64 (2.99)	6.47 (5.52)	5.86 (7.37)	0.481	0.016
Education		2.48 (2.18)	4.48 (3.76)	3.17 (2.92)	0.080	0.055
National Presentations						
Total	31	3.60 (2.68)	5.68 (5.18)	5.24 (7.16)	0.397	0.020
Education		2.24 (2.04)	3.98 (3.05)	2.63 (2.38)	**0.035**	0.072
International Presentations						
Total	31	0.62 (0.93)	0.79 (1.15)	0.74 (1.11)	0.813	0.005
Education		0.17 (0.29)	0.48 (0.86)	0.43 (0.59)	0.093	0.051
Leadership Roles						
Total	31	0.77 (0.72)	1.26 (1.12)	0.92 (0.60)	0.556	0.013
Education		0.61 (0.64)	0.98 (1.10)	0.71 (0.61)	0.739	0.007

^a^Abbreviations: Macy Faculty Scholars Program (MFSP)

^b^Between-group differences were tested with a one-way analysis of variance (ANOVA) test

^c^Rates of accomplishment were expressed as numbers per year

^d^Effect sizes were estimated with a partial eta-squared test, which attributes the variation in a given output variable based on a certain explanatory variable and that is not explained by another variable in the model

^e^Five Scholars were excluded from the grant dollars analysis given lack of grant funding

Three areas of accomplishment demonstrated statistically significant temporal differences, though with different patterns of change. The rate of education-specific grants (in principal investigator role) decreased going from time period 1 to 2 as well as from time period 2 to 3. The rate of total grant dollars (either in a PI or co-investigator role) decreased going from time period 1 to 2 but increased going from time period 2 to 3. The rate of education-specific national presentations increased going from time period 1 to 2 but decreased going from time period 2 to 3.

Four areas demonstrated a non-statistically significant trend toward temporal differences. The rate of education-specific grants (either in a PI or co-investigator role) decreased going from time period 1 to 2 as well as from time period 2 to 3. The rates of total publications, education-specific presentations (any regional, national, and international), and education-specific international presentations increased going from time period 1 to 2 but decreased going from time period 2 to 3.

Table 3 details a post-hoc analysis to assess the effect of protected time on the rate of accomplishments, and demonstrated total grants, total grants as PI, education-specific grants, education-specific grants as PI, education-specific publications, and education-specific last author publications all had statistically significant correlation with the amount of protected time.

**Table 3:** Effect of protected time on outcomes of the Macy Foundation Scholar Program^a^

Accomplishment	*P*-value	Partial Eta Squared	Observed Power	Adjusted R Squared
Honors				
Total	0.513	0.047	0.204	-0.014
Education	0.579	0.041	0.179	-0.021
Grants				
Total	0.050	0.151	0.637	0.097
Education	0.032	0.169	0.699	0.116
Grants as PI				
Total	0.017	0.194	0.780	0.143
Education	0.019	0.190	0.767	0.138
Grant Dollars				
Total	0.151	0.106	0.449	0.048
Education	0.475	0.051	0.220	-0.009
Grant Dollars as PI				
Total	0.338	0.069	0.290	0.009
Education	0.253	0.082	0.348	0.024
Publications				
Total	0.043	0.158	0.660	0.104
Education	0.003	0.252	0.909	0.205
First Author Publications				
Total	0.381	0.063	0.266	0.003
Education	0.396	0.061	0.257	0.001
Last Author Publications				
Total	0.210	0.091	0.386	0.033
Education	0.023	0.182	0.743	0.13
All Presentations				
Total	0.520	0.046	0.201	-0.014
Education	0.128	0.113	0.481	0.056
National Presentations				
Total	0.447	0.054	0.233	-0.006
Education	0.282	0.077	0.327	0.018
International Presentations				
Total	0.841	0.017	0.099	-0.045
Education	0.074	0.136	0.577	0.081
Leadership Roles				
Total	0.881	0.014	0.089	-0.049
Education	0.461	0.053	0.226	-0.008

^a^Authors utilized ANCOVA Model with protected time as covariate

Survey response rate was 94% (34/36). Most respondents “agreed” or “strongly agreed” that the MFSP developed them as a teacher (29/34), mentor (27/34), scholar (33/34), and educational leader (34/34). Most “agreed” or “strongly agreed” that the program was a valuable use of time (33/34), allowed things to be accomplished that otherwise would not have been (34/34), and would recommend the program to others (33/34). Scholars reported a mean and median of 51 ± 12% protected time.

### Qualitative analysis

The main themes identified in the qualitative analysis provide insight into the *process* component of the CIPP model and fit under two overarching domains: areas of strength and areas for improvement (Table 4). Themes related to strengths included: mentorship, community of practice, and resources. Themes related to areas for improvement included: lack of diversity; technology; and individualization.

**Table 4:** Qualitative analysis of survey responses

Areas of strength		
**Themes**	**Codes**^**a**^	**Representative Quotes**
**Mentorship**	Mentorship (2a, 3)	“Mentorship. The quality of people I have met in the MFSP is extraordinary. I feel very very fortunate.” “The world renowned mentors who share their time and expertise to make us better.”
	Sponsors (2a)	“Sponsorship: They want us to succeed. They include us in programming and recommend us for positions.”
**Community of Practice**	Networking (2a, 3, 8)	“It is a unique network of scholars dedicated to health professions education.” “Opportunity to network with colleagues doing cutting edge educational work.”
	Connection (2a, 3)	“Connection with national peers and leaders.”
	Cohort (2a)	“Cohort/Cadre of educational scholars.” “Colleagues, colleagues, colleagues–friends for life.”
	Career Development (3, 7, 11)	“The MFSP is the most impactful career development program I have ever seen. It is unique in all of health professions education and fills a profound gap. It was career changing for me. I simply would not have the career that I have today without it.”
**Resources**	Protected Time (2a, 8)	“This gave me the TIME to do the program I needed to do including the research.” “Adequate amount of protected time to develop an area of program development and scholarship in a novel area of medical education.”
	Financial Support (2a)	“The financial assistance for traveling to meetings gave me unprecedented access to meetings that I wouldn't have been able to attend otherwise.” “Financial support to pursue educational work that is not otherwise generally supported in academia.”
	Leverage (2a, 8)	“Provide prestige and validation to be considered for future leadership roles.” “Validation as a leader from a highly reputable national organization.” “Increased visibility at my home institution and nationally—I strongly believe this award ‘opened doors’ for me in my career.”
	Curriculum (2a, 3)	“Incorporation of Harvard Macy program for educators (for me as someone who previously did not have formal med ed training, this was incredibly useful and served as a foundation for my med ed work).”
**Areas for improvement**		
**Themes**	**Codes**	**Representative Quotes**
**Lack of Diversity**	Professional Diversity (2b, 3)	“Greater professional diversity.” “Heavy on medicine.”
	Scholar Diversity (2b)	“Greater attention to equity among scholars, mentors, program goals and process.”
	Mentor Diversity (2b, 3)	“Diversification of Macy Advisory Committee members.”
	Health Equity and Disparities (2b)	“Advancing health equity.”
**Technology**	Social Media Presence (2b)	“Using the website and social media to keep us up-to-date or the work and accomplishment of scholars.”
	Remote Learning (2b)	“More contacts through Zoom platform throughout the year.”
**Individualization**	Flexibility (2b)	“Have more flexibility with use of the funding.”
	Grant Writing (2b)	“NIH grant writing support.” “Support to develop research proposals for AHRQ.”
	Personal Coaching (2b)	“Consider offering continued formalized leadership growth opportunities for former MFS.” “Could there be coaching available for the Scholars? This could be something that Scholars could apply for. Could provide deep transformative guidance.”
	Alumni Connection (2b, 3)	“Transition out of the program.” “Continuous programming and connection beyond two year fellowship.” “Further emphasis on transition from Macy Faculty Scholars program to next phase–maybe emphasizing inter cohort communication outside of the annual meeting.”
	Standardization of Mentorship (2b, 3)	“Mentorship varies tremendously.” “Better defined expectations and outcomes for mentor/mentee relationship with Macy-assigned mentors.” “There are no standard expectations for the national mentor, for example, my mentor did not reach out to me and often did not respond to my request for mentorship. Some standardization and transparency would be appreciated.”

^**a**^Listed under each code in parentheses represents the survey questions corresponding to each code

## Discussion

We utilized the CIPP framework to complete a robust, academically rigorous, program evaluation of a national FD program to add and further advance the literature supporting the need to invest in health profession educators. Our data demonstrated that the participants of this program were developed as mentors, scholars, and educational leaders, indicating a successful interplay of the CIPP framework components: context, inputs and process generating intended products. In addition to a program evaluation of the MFSP, our data provide additional information for FD leaders to help design successful programs by highlighting the importance of protected time on increased scholarly activity and how mentorship, CoPs, resources, and diversity serve as key components of all FD programs.

### Context

In the CIPP model, the c*ontext* is defined as the program objectives, the basis that supports those objectives, and the learner identification and demand [19, 20]. To understand the context of the MFSP program, we analyzed the MFSP program description. Scholars accepted to the MFSP program receive 50% protected time for two years during the program. The expectation is that Scholars produce academically, are engaged in the MFSP CoP, and participate in the Harvard Macy Institute Program for Educators in Health Professions throughout that time period [12]. This context positively affected career growth and academic output for the Scholars as demonstrated through increased academic output of publications and presentations during the program years.

### Inputs

The *inputs* of the CIPP model are described as the assessment of the educational strategies employed to meet the objectives, including program plans and resources. The MFSP scholars represent key inputs as they form a CoP. Regarding Scholar demographics, our data demonstrated diversity in gender, race, professional degree, and academic rank of the Scholars accepted to the program. In our analysis, the percentage of Scholars identifying as women matched the percentage of women applicants. Furthermore, the percentage of underrepresented in medicine (URM) Scholars represented double the percentage of URM applicants. Lack of diversity in the health professions remains a critically important issue with evidence that diversity within the care team enhances patient care [25–32]. In addition, FD opportunities for URM and women health professionals enhance retention and advancement of these same groups of faculty [33–37]. By continuing to emphasize recruitment of URM Scholars and mentors to the MFSP, and focusing curricular interventions on addressing implicit bias, social determinants of health, health care disparities, and systemic racism within medical education, FD programs can better meet their goal of training Scholars to drive critical change.

The MFSP utilized several educational strategies to meet the previously described objectives. First, they utilized their national network of mentors, faculty, and alumni to create a robust CoP that facilitated career growth and academic output for the Scholars. Second, the program accessed the Harvard Macy Institute Program for Educators in Health Professions curriculum which allowed the Scholars to participate in an established program that guided their own professional development as health professions education scholars. The MFSP use of these resources and educational strategies supported the program’s intended goals, and the actual implementation was further explored as part of the *process* analysis.

### Process

Regarding the *process* of the CIPP model, or the actual implementation and execution of the planned activities to support the *products*, we found that the MFSP program was ultimately successful in its implementation and execution of planned activities. Our data demonstrated successful mentorship relationships, robust CoP, significant resources, and growth as educational scholars. While the participants rated the program highly overall, the analysis also revealed specific areas for improvement, including recommendations regarding mentorship, diversity, technology, and a request for continued engagement in the program post-graduation.

Mentorship and sponsorship in academic medicine represent key drivers of personal and professional development, research productivity, and career satisfaction [38–40]. Although most Scholars rated mentorship and sponsorship highly, many Scholars noted a lack of standardization of mentorship. Although the MFSP included nationally recognized, prolific scholars as mentors, many had participated in the program since its inception, which limited the diversity of perspective and also potentially contributed to a lack of diversity among the MFSP program faculty, as mentioned by the Scholars. One resultant suggestion for the MFSP and similar FD programs would be to have mentors fill a limited-service term before allowing new mentors to enter. Additionally, allowing mentees to select their own mentors, ensuring incorporation of components of formal mentoring programs, encouraging a written mentor–mentee contract, and exploring alternate models for mentorship arrangements represent potential future strategies previously described [39].

In addition, Scholars indicated that the MFSP afforded entry into a robust CoP of educators in the health professions, including other Scholars, alumni, and faculty of the program. Membership in CoPs result in increased social support, which can enhance well-being and engagement, and may mitigate the risk of burnout [41–43]. Also, Scholars noted resources to be a strength of the MFSP as protected time and financial support facilitated productivity by addressing a common barrier to scholarly work [8]. The majority of Scholars reported that they did achieve the intended 50% protected time. Although participation in FD programs often comes with the promise of “protected time,” the degree to which it materializes varies. Even with funding, clinical and other administrative demands can easily consume time intended for academic pursuits, particularly for clinician-educators [44, 45]. Without truly protected time, faculty must utilize evening and weekend hours to accomplish scholarly and professional development activities, with weekend activities being a driver of burnout [46]. The compelling finding that Scholars largely received the amount of protected time promised likely contributed significantly to the products of the program and should be duplicated in other FD programs to achieve similar successes in scholarly activity.

Finally, integration of technology represents another potential area of growth for the MFSP and FD programs in general. Specifically, Scholars suggested improving the MFSP’s social media presence and increasing the frequency of contact via videoconferencing and other digital platforms, a theme further highlighted in the context of COVID-19 related gathering restrictions. Furthermore, Scholars requested continuous programming and connection to the MFSP community beyond the two-year fellowship by enhancing the alumni network and promoting communication in between in-person meetings. Engaging past participants of FD programs as future leaders of the program enlarges its CoP, while also providing future sponsorship and leadership opportunities to HPEs. Utilizing these approaches allow for affordable and flexible ways to promote engagement among geographically distant participants and enhance a CoP [47–49].

### Products

The *products* of the CIPP model, the resulting outcomes or achievements of the program, demonstrated that the MFSP successfully developed its Scholars academically. Prior studies have evaluated competitive grant awards and publications as indicators of academic success among faculty and we also utilized these metrics to assess that objective [36, 50–54]. We found that education publications and presentations increased during and following MFSP participation, suggesting that the Scholars were disseminating their research and innovations in education. The pronounced increase in publications and presentations during the program years likely indicate the particularly beneficial effect of protected time. Of note, there was a decrease in productivity observed when moving from time in the program to time outside of the program which likely reflects the challenges with the ‘transition back’ from a significant proportion of protected time to typical faculty roles. Prior literature has found that individual priorities and work environment characteristics such as number of work hours, quality of mentorship relationships, and institutional support augment success with this transition [55]. Ongoing mentorship and participation in the CoP through the MFSP alumni status and creative efforts by individual institutions to provide ongoing protected time for productivity and development beyond the completion of the program may sustain benefits of participation [55, 56]. Indeed, the amount of protected time offered by the MFSP distinguishes it among FD programs for health professions educators; yet, this finding highlights the continued need for FD programs to support protected time, while adding to the body of literature demonstrating the meaningful outcomes generated by such an investment.

### Implications of program evaluation findings

As the MFSP represents a flagship FD program for health professions educators, lessons learned through its evaluation provide further evidence for policymakers, organizational and institutional leaders that investing in health professions educators, particularly through providing actualized protected time, provides meaningful return on investment with regard to developing leaders, scholars, teachers, and mentors, with the intent of benefiting society at large. Additionally, the program evaluation generated a list of key themes and resultant strategies to inform future iterations of the MFSP program that are broadly applicable to other faculty developers in health professions education as they create, optimize, and expand their programs (Table 5).

**Table 5:** Recommendations for faculty development programs

1. Maintain investment in program resources
• Provide financial support to protect time for faculty • Provide ongoing opportunities for networking and community-of-practice building • Prioritize excellent mentorship and sponsorship of faculty • Communicate prestige of the program such that faculty can benefit from the leverage gained by their participation
2. Focus on diversity within multiple domains
• Push for increased representation of women and URM faculty • For external programs, recruit faculty from geographic areas not yet represented by prior cohorts • Recruit faculty from other health professions outside of medicine and nursing • Ensure diversity of program faculty/mentors (including gender, URM status, and health profession type) and be mindful of alignment with participant demographics • Recruit applicants with innovative projects related to diversity, equity, and inclusion within the health professions • Emphasize curricular interventions that teach faculty to combat implicit bias, health care disparities, social determinants of health and systemic racism
3. Optimize mentorship
• Provide faculty with options for mentors and allow them autonomy in selecting a mentor • Ensure components of a formal mentoring program are incorporated: encourage a written mentor–mentee contract; provide a suggested “roadmap” of mentor activities including the ideal number and frequency of mentor–mentee check ins; provide an explicit description of mentor expectations • Explore alternate models for mentorship arrangements beyond dyads (speed mentoring, peer, facilitated peer, etc.) • Collect real-time feedback from faculty during the program about the quality of mentors; provide the opportunity to be paired with additional or alternative mentors if needed • Consider use of an evidence-based mentorship assessment tool such as the Scholarly Teaching in Health Professions Education (STHPE) to document effectiveness and quality of mentorship [57] • Use data to establish mentorship standards and target faculty development of mentors (such as through remote video conferencing sessions about mentorship best practices) • Formally acknowledge exemplary mentors, such as through a “mentor of the year award,” in order to reinforce and encourage excellence in mentorship
4. Leverage technology to enhance the programs’ community of practice for current and alumni members
• Utilize social media to amplify faculty success by tweeting about their publications and accomplishments, tweet pearls from educational sessions, tweet about blog posts, follow Twitter accounts of faculty, mentors, and potential applicants • Utilize platforms such as email listservs, closed Facebook groups or social network enterprises (i.e. Slack, Microsoft Teams or Basecamp) to provide a virtual “space” for faculty to communicate, interact socially, share resources and collaborate • Implement regular (at least monthly) remote check-ins via videoconferencing platforms (i.e., Zoom, WebEx, Blue Jeans) to promote social interactions, increase contact among members of the MFSP community, provide a forum to discuss educational topics relevant to stakeholders, and/or serve as writing accountability groups • Invite alumni to partake in the previously described activities to enhance alumni connection to the program’s network and continue professional development beyond the two-year period • Maintain an online database of faculty and alumni interests to facilitate collaboration within the community and to offer speaking and writing opportunities
5. Optimize the curriculum
• Perform an individual needs assessment for each faculty at the beginning of the program and allow faculty flexibility with curricular components according to individual needs identified during this process • Include curriculum on diversity, equity, and inclusion with attention paid to credibility of faculty chosen to deliver said curriculum • Ensure educational sessions for faculty are interactive and learner-centered rather than lecture-based • Integrate a coaching program focused on enhancing faculty leadership skills • Provide targeted curricular interventions to improve faculty knowledge, skills and comfort with regard to grant writing

In applying the lessons derived from the program evaluation, however, the learning and practice environments of related FD programs must be taken into consideration. The MFSP is a premier, nationally recognized program that draws faculty learners from institutions throughout the country. There are few similar programs. Instead, most longitudinal FD programs draw from, and are supported by, single institutions which seek to build their local educational expertise. The contrast between the national focus of the MFSP and the local focus of most institutional programs is significant in many ways. The financial support for protected time is critical to the MFSP but “support” from local programs may well reflect the influence of other values and priorities to foster the strength and reputation of the institution – above and beyond the finances for “protected time.”

Similarly, the MFSP seeks to build a national community of scholars and collaborators, whereas local programs are likely to be more concerned with building their own, local CoP. There is a risk of a local FD program focusing too narrowly on the needs and benefits of the institution but we, the authors, many of whom have participated in local FD programs, believe firmly that all local programs should recognize that medical education is a national, even international, enterprise that extends beyond the narrower interests of local contexts.

Finally, we believe that all longitudinal FD programs should rigorously evaluate themselves for both local impact but also national significance. The perspective of evaluation needs to be national in scope and simply describing a program is only a first step towards the needed analysis of the key features of the program, the characteristics of the learners, and the nature of the educational context. Leaders of such FD programs need to build the community’s knowledge and practice, not just a local institution. This is how individual programs contribute to the greater good in health professions education.

## Limitations

We acknowledge several limitations of our evaluation. Of note, over the 10-year period examined, the curriculum changed dynamically in response to ongoing feedback, such that Scholars' experiences varied depending on the years they participated. Additionally, CVs vary in their format and content, and different reviewers vary in their CV interpretation. In order to streamline data extraction, the CV analysis subgroup jointly coded a CV in order to develop the extraction form (see Additional file 1: Appendix 2) through an iterative process that generated qualifying footnotes for various categories and involved real-time communication to resolve discrepancies. Ideally, the evaluation would have been strengthened by obtaining CV data from faculty not selected to serve as a control group, accessing data from non-Scholar stakeholders (i.e., medical school deans, mentors, mentees), and analyzing program costs. Ultimately, we chose the more feasible approach of surveying Scholars and using them as their own individual controls by comparing an equivalent number of years of productivity during pre- and post- time periods. Thus, we evaluated a longer time period for the earlier Scholar cohorts and a shorter time period for later Scholars, which could potentially skew results. Although a more resource-intensive approach, conducting focus groups or interviews would have greatly enhanced the richness of data generated around the scholars’ experiences with program implementation, and the authors recommend for those considering embarking on robust FD program evaluation to incorporate these methods if feasible. Additionally, many other external confounding factors likely contributed to differences between the time periods.

Of note, the large number of statistical comparisons could technically lead to spurious results for the quantitative data. Although conservative measures such as Bonferroni corrections were considered, we withheld use of them, given that the trends were of greater practical concern for the program evaluation than statistical significance. In addition, trends were also supported by themes identified by a separate review group in the qualitative analysis data.

Despite these limitations, the evaluation process’ many strengths included: multiple independent and objective perspectives of early career health professions educators who represent potential stakeholders, a detailed analysis of Scholars’ CVs, a relatively high response rate to the survey administered, use of an applicable evaluation framework previously utilized for FD programs, and integration of findings with relevant education theory and literature.

## Conclusions

Over the 10-year course of its history, the MFSP has received positive responses from participants and has successfully developed Scholars as mentors, teachers, educators, and scholars. The increase in academic productivity observed most prominently during the two-year period of the program highlights the importance of investing in health professions education faculty through actualized, rather than simply promised, protected time. Scholars also commonly cited the mentorship and CoP as the most valuable components of the program for achieving its stated goals. Areas for improvement included enhancing the diversity of program participants, program leaders and mentors across multiple domains including race, gender, geographic location, and health profession type; leveraging technology to strengthen the MFSP CoP through increased social media presence and use of remote teaching strategies; and improving flexibility of the program to meet Scholar needs. Through this program evaluation, we have demonstrated key strategies that can be utilized by other existing FD programs to inform continued development and expansion of their programs and ultimately, support the important work and development of health profession educators.

## Supplementary Information

**Additional file 1:** **Appendix 1. **CV Data Extraction Form. **Appendix 2. **MFSP Scholar Survey.

## Data Availability

The datasets generated and/or analyzed during the current study are not publicly available because the Macy Foundation is a private foundation but are available from the corresponding author on reasonable request.

## References

[1] Guskey TR (2003). What Makes Professional Development Effective?. Phi Delta Kappan.

[2] Steinert Y, Dent JA, Harden RM, Hunt D, Hodges BD (2017). Staff Development. A practical guide for medical teachers.

[3] Sheets KJ, Schwenk TL (1990). Faculty development for family medicine educators: An agenda for future activities. Teach Learn Med.

[4] Sethi A, Schofield S, Ajjawi R, McAleer S (2016). How do postgraduate qualifications in medical education impact on health professionals?. Med Teach.

[5] Hoy WK, Miskel CG, Tarter CJ (2012). Educational administration: theory, research, and practice.

[6] Steinert Y, McLeod PJ, Boillat M, Meterissian S, Elizov M, Macdonald ME (2009). Faculty development: a ‘Field of dreams’?. Med Educ.

[7] Steinert Y, Nasmith L, McLeod PJ, Conochie L (2003). A teaching scholars program to develop leaders in medical education. Acad Med.

[8] Zibrowski EM, Weston WW, Goldszmidt MA (2008). ‘I don’t have time’: issues of fragmentation, prioritisation and motivation for education scholarship among medical faculty. Med Educ.

[9] Rushmer R, Kelly D, Lough M, Wilkinson JE, Davies HTO (2004). Introducing the learning practice – II. Becoming a learning practice. J Eval Clin Pract.

[10] Onyura B, Ng SL, Baker LR, Lieff S, Millar B-A, Mori B (2017). A mandala of faculty development: using theory-based evaluation to explore contexts, mechanisms and outcomes. Adv Health Sci Educ.

[11] Macy Faculty Scholars. Josiah Macy Jr Foundation. https://macyfoundation.org/macy-scholars. Accessed 20 May 2021.

[12] Harvard Macy Institute. https://harvardmacy.org/index.php.

[13] Leslie K, Baker L, Egan-Lee E, Esdaile M, Reeves S (2013). Advancing faculty development in medical education: a systematic review. Acad Med.

[14] Thompson BM, Searle NS, Gruppen LD, Hatem CJ, Nelson E (2011). A national survey of medical education fellowships. Med Educ Online.

[15] Steinert Y, Mann K, Centeno A, Dolmans D, Spencer J, Gelula M (2006). A systematic review of faculty development initiatives designed to improve teaching effectiveness in medical education: BEME Guide No. 8. Med Teach.

[16] Onyura B (2020). Useful to whom? Evaluation utilisation theory and boundaries for programme evaluation scope. Med Educ.

[17] Carraccio C, Englander R, Van Melle E, ten Cate O, Lockyer J, Chan M-K (2016). Advancing competency-based medical education: a charter for clinician-educators. Acad Med.

[18] Sklar DP, Weinstein DF, Carline JD, Durning SJ (2017). Developing programs that will change health professions education and practice: principles of program evaluation scholarship. Acad Med.

[19] Stufflebeam DL, Shinkfield AJ (2007). Evaluation theory, models, and applications.

[20] Steinert Y (2000). Faculty development in the new millennium: key challenges and future directions. Med Teach.

[21] Frye AW, Hemmer PA (2012). Program evaluation models and related theories: AMEE guide no. 67. Med Teach.

[22] Stufflebeam D (1983). Program Evaluation. Evaluation Models.

[23] Popham WJ (1993). Educational evaluation.

[24] O’Brien BC, Harris IB, Beckman TJ, Reed DA, Cook DA (2014). Standards for reporting qualitative research: a synthesis of recommendations. Acad Med.

[25] Yu PT, Parsa PV, Hassanein O, Rogers SO, Chang DC (2013). Minorities struggle to advance in academic medicine: A 12-y review of diversity at the highest levels of America’s teaching institutions. J Surg Res.

[26] Karani R, Varpio L, May W, Horsley T, Chenault J, Miller KH (2017). Commentary: racism and bias in health professions education. Acad Med.

[27] Association of American Medical Colleges (2019). Diversity in Medicine: Facts and Figures 2019.

[28] Agency for Healthcare Research and Quality (2017). National Healthcare Disparities Report.

[29] Brown DJ, DeCorse-Johnson AL, Irving-Ray M, Wu WW (2005). Performance evaluation for diversity programs. Policy Polit Nurs Pract.

[30] Grumbach K, Mendoza R (2008). Disparities in human resources: addressing the lack of diversity in the health professions. Health Aff (Millwood).

[31] Health Resources and Services Administration. The rationale for diversity in the health professions: a review of the evidence. Rockville, MD: U.S. DHHS; 2006.

[32] Smedley BD, Butler A, Bristow L (2004). In the Nation’s Compelling Interest: Ensuring Diversity in the Health-Care Workforce.

[33] Daley S, Wingard DL, Reznik V (2006). Improving the retention of underrepresented minority faculty in academic medicine. J Natl Med Assoc.

[34] Rodriguez JE, Campbell KM, Fogarty JP, Williams RL (2014). Underrepresented minority faculty in academic medicine: a systematic review of URM faculty development. Fam Med.

[35] Chang S, Guindani M, Morahan P, Magrane D, Newbill S, Helitzer D (2020). Increasing promotion of women faculty in academic medicine: impact of national career development programs. J Womens Health.

[36] Rust G, Taylor V, Herbert-Carter J, Smith QT, Earles K, Kondwani K (2006). The Morehouse faculty development program: evolving methods and 10-year outcomes. Fam Med.

[37] Guglielmo BJ, Edwards DJ, Franks AS, Naughton CA, Schonder KS, Stamm PL (2011). A critical appraisal of and recommendations for faculty development. Am J Pharm Educ.

[38] Sambunjak D, Straus SE, Marušić A (2006). Mentoring in Academic Medicine: A Systematic Review. JAMA.

[39] Kashiwagi DT, Varkey P, Cook DA (2013). Mentoring programs for physicians in academic medicine: a systematic review. Acad Med.

[40] Ayyala MS, Skarupski K, Bodurtha JN, González-Fernández M, Ishii LE, Fivush B (2019). Mentorship is not enough: exploring sponsorship and its role in career advancement in academic medicine. Acad Med.

[41] Bartle E, Thistlethwaite J (2014). Becoming a medical educator: motivation, socialisation and navigation. BMC Med Educ.

[42] Maslach C, Schaufeli WB, Leiter MP (2001). Job Burnout Annu Rev Psychol.

[43] Glew RH, Russell JC (2013). The importance of community in academic health centers. Teach Learn Med.

[44] Sheffield JV, Wipf JE, Buchwald D (1998). Work activities of clinician-educators. J Gen Intern Med.

[45] Beasley BW, Wright SM (2003). Looking forward to promotion: characteristics of participants in the prospective study of promotion in academia. J Gen Intern Med.

[46] West CP, Dyrbye LN, Shanafelt TD (2018). Physician burnout: contributors, consequences and solutions. J Intern Med.

[47] Cahn PS, Benjamin EJ, Shanahan CW (2013). ‘Uncrunching’ time: medical schools’ use of social media for faculty development. Med Educ Online.

[48] Klein M, Niebuhr V, D’Alessandro D (2013). Innovative online faculty development utilizing the power of social media. Acad Pediatr.

[49] Chan TM, Gottlieb M, Sherbino J, Cooney R, Boysen-Osborn M, Swaminathan A (2018). The ALiEM faculty incubator: a novel online approach to faculty development in education scholarship. Acad Med.

[50] Roy KM, Roberts MC, Stewart PK (2006). Research productivity and academic lineage in clinical psychology: who is training the faculty to do research?. J Clin Psychol.

[51] Sax LJ, Hagedorn LS, Arredondo M, Dicrisi FA (2002). Faculty research productivity: exploring the role of gender and family-related factors. Res High Educ.

[52] Eisen A, Barlett P (2006). The Piedmont project: fostering faculty development toward sustainability. J Environ Educ.

[53] Karimi R, Arendt CS, Cawley P, Buhler AV, Elbarbry F, Roberts SC (2010). Learning bridge: curricular integration of didactic and experiential education. Am J Pharm Educ.

[54] Nunez-Wolff C (2007). A study of the relationship of external funding to medical school faculty success. Diss Abstr Int Sect Humanit Soc Sci.

[55] Jagsi R, Griffith KA, Jones RD, Stewart A, Ubel PA (2017). Factors associated with success of clinician-researchers receiving career development awards from the national institutes of health: a longitudinal cohort study. Acad Med.

[56] Anderson CM, Campbell J, Grady P, Ladden M, McBride AB, Montano NP (2020). Transitioning back to faculty roles after being a Robert Wood Johnson Foundation Nurse Faculty scholar: challenges and opportunities. J Prof Nurs Off J Am Assoc Coll Nurs.

[57] St-Onge C, Young M, Varpio L (2019). Development and validation of a health profession education-focused scholarly mentorship assessment tool. Perspect Med Educ.

