# Correction to: A decade of faculty development for health professions educators: lessons learned from the Macy Faculty Scholars Program

**DOI:** 10.1186/s12909-023-04256-7

**Published:** 2023-05-01

**Authors:** Mary Haas, Justin Triemstra, Marty Tam, Katie Neuendorf, Katherine Reckelhoff, Rachel Gottlieb-Smith, Ryan Pedigo, Suzy McTaggart, John Vasquez, Edward M. Hundert, Bobbie Berkowitz, Holly J. Humphrey, Larry D. Gruppen

**Affiliations:** 1 Department of Emergency Medicine, University of Michigan Medical School, Ann Arbor, MI USA; 2 Department of Pediatrics and Human Development, Corewell Health, Helen DeVos Children’s Hospital, Michigan State University College of Human Medicine, Grand Rapids, MI USA; 3 Division of Cardiovascular Medicine, University of Michigan, Ann Arbor, MI USA; 4 Department of Palliative and Supportive Care, Cleveland Clinic Lerner College of Medicine of Case Western Reserve University, Cleveland, OH USA; 5 College of Chiropractic, Cleveland University, Kansas City, Overland Park, KS USA; 6 School of Medical & Applied Sciences, Central Queensland University, Brisbane City, QLD Australia; 7 Department of Pediatrics, University of Michigan Medical School, Ann Arbor, MI USA; 8 Department of Emergency Medicine, Harbor-UCLA Medical Center, Emergency Medicine, David Geffen School of Medicine at UCLA, Los Angeles, LA USA; 9 Office of Medical Student Education, University of Michigan Medical School, Ann Arbor, MI USA; 10 Arapahoe Community College, Littleton, CO USA; 11 Medical Education, Harvard University, Harvard Medical School, Boston, MA USA; 12 Columbia University School of Nursing and University of Washington School of Nursing, Seattle, WA USA; 13 Josiah Macy, Jr. Foundation, New York, NY USA; 14 The University of Chicago, Chicago, IL USA; 15 Department of Learning Health Sciences, University of Michigan Medical School, Ann Arbor, MI USA

**Correction: BMC Medical Education (2023) 23:185**

10.1186/s12909-023-04155-x

Following publication of the original article [1], we have been informed to delete Daniel D. Federman and Ralph W. Gerard as authors as they did not contribute to this paper. Drs. Federman and Gerard were not included in the original submission by the authors and were entered in error during the publishing process.

Also, we have updated the affiliation of author Holly J. Humphrey as follows:

From: Josiah Macy, Jr. Foundation, New York, NY, USA.

To: Josiah Macy, Jr. Foundation, New York, NY, USA.

The University of Chicago, Chicago, IL, USA.

The authorship has been updated above and the original article has been corrected. The publisher apologizes for this error.
