# Supplementary material for: A decade of faculty development for health professions educators: lessons learned from the Macy Faculty Scholars Program
Source: BMC Med Educ. 2023 Mar 27;23:185. doi: 10.1186/s12909-023-04155-x (PMC10041479; doi:10.1186/s12909-023-04155-x)
Supplement: Supplementary file 1 — Additional file 1: Appendix 1. CV Data Extraction Form. Appendix 2. MFSP Scholar Survey. [file 12909_2023_4155_MOESM1_ESM.docx]

**Supplemental Digital Appendix 1. CV Data Extraction Form**

CV Data Extraction form

(Please follow the footnotes for guidance in completing the form)

Scholar Name: ___________________________________________________

Time period 1 (Pre-MFSP) __________ to __________

Time period 2 (Intra-MFSP, 2-year period starting the year after admission ______ to ______

Time period 3 (Post-MFSP) __________ to __________

Outcome Time Period 1 Time Period 2 Time period 3

New^1^ honors:

#All types __________ __________ __________

#Education only __________ __________ __________

New^1^ grant funding:

#All types __________ __________ __________

#PI role^2^ __________ __________ __________

Total $^3^ __________ __________ __________

All PI $^4^ __________ __________ __________

#Education only __________ __________ __________

#PI role^2^ __________ __________ __________

Total $^3^ __________ __________ __________

Ed PI $^5^

Peer-reviewed publications & book chapters:^6^

#All __________ __________ __________

#first (or sole) author __________ __________ __________

#senior (last) author __________ __________ __________

#Education only

(include QI/patient safety^7^) __________ __________ __________

#first (or sole) author __________ __________ __________

#senior (last) author __________ __________ __________

Outcome Time Period 1 Time Period 2 Time period 3

Presentations:^8^

#All^9^ __________ __________ __________

#national __________ __________ __________

#international __________ __________ __________

#Education only^7^

(include QI/patient safety^5^) __________ __________ __________

#national __________ __________ __________

#international __________ __________ __________

New^1^ leadership roles/titles^10^

#All __________ __________ __________

#Education only^11^ __________ __________ __________

New^1^ promotions^12^ __________ __________ __________

Move to a new institution __________ __________ __________

# New sponsored/named

professorships received __________ __________ __________

Notes and questions to follow-up:

^1^ Use starting date to assign to time period 1, 2 or 3

“Awards” may be both honorific awards and funding awards. Be sure to code them into the correct outcome.

Do NOT code Macy Faculty Scholars Program participation – individuals variously categorize them as ‘honors,’ ‘grants,’ or other outcomes.

^2^ Include any PI title, excl. investigator and co-investigator

^3^ if no $ amounts provided, or presented as %FTE, do not include

^4^ Total of all grants on which the Scholar is PI, co-PI, collaborating PI, any other PI – ignore grant $ if Scholar is investigator or other role.

^5^ Total of educational grants on which the Scholar is PI, co-PI, collaborating PI, any other PI – ignore grant $ if Scholar is investigator or other role.

^6^ Peer-reviewed only, but include book chapters

^7^ Not just any patient or clinical care topic but specifically on quality improvement or patient safety initiatives/studies.

^8^ Include workshops, posters, invited lectures, blogs, abstracts – whatever the participant includes on their CV.

^9^ Exclude all local (intramural) presentations

^10^ Include chair, chief, president, secretary, director, etc. Exclude educational roles, like mentor, advisor, etc.

^11^ Recognizing that many administrative roles include some educational responsibilities (e.g., residency program director), if it contains a meaningful (to you) amount of educational leadership, include it in the education only count.

^12^ Academic track (professorial) only, not administrative promotions

**Supplemental Digital Appendix 2. MFSP Scholar Survey**

As the Macy Foundation selects the 10th cohort of the Macy Faculty Scholars Program it wishes to evaluate the program for possible improvements and to ensure it addresses the Foundation’s goals. The Foundation has commissioned a group of independent evaluators to gather and interpret it. Your responses will remain confidential and shared with the Foundation only in aggregate form.

Please evaluate your experience in the Macy Foundation Scholarly Program (MFSP) by responding to the questions and statements below. Your responses will be very valuable in the continuing design and evaluation of this program.

Thank you!

1. In my experience, the MFSP… (answer choices: strongly disagree, disagree, neutral, agree, strongly agree):

- developed me as a teacher
- developed me as a mentor
- developed me as scholar
- developed me as an educational leader
- was a valuable use of my time
- allowed me to accomplish things I would otherwise not have accomplished
- is a program I would recommend to my colleagues

2a. Please describe three strengths of the MFS program (Type as much as you need:

Strength #1:

Strength #2:

Strength #3:

2b. Please describe three areas for improvement of the MFS Program (Type as much as you need):

Area for improvement #1:

Area for improvement #2:

Area for Improvement #3:

3. In terms of your experience with the specific MFSP curriculum components listed below, please describe its strengths, areas for improvement, or if you did not participate in it. Type as much as you need. Text boxes for each row included: Strengths, Areas for Improvement, Did not participate

Harvard Macy Institute:

Skeff Faculty Development Program:

MFSP Mentoring:

Local Mentoring:

4. On average, how much protected time did you have to work on the MFSP, including your project? Please reply with the percent of Full-Time Equivalent (% FTE) (Sliding bar with 0-100%)

5. How did you obtain the time? Select all that apply: reduced clinical time, reduced/gave up other educational responsibilities, reduced/gave up other non-educational work responsibilities, reduced/gave up personal/family responsibilities, increased my overall work hours, other

6. Which educational program(s) did you start during your time as a scholar? Which are still running?

7. Could you have completed your project without participating in the MFSP? Please explain:

8. Since your induction into the MFSP program, what accomplishment are you most proud of and why? How did your participation in MFSP contribute to your accomplishments?

9. Since your completion of MFSP, how has your conduct of health professions education contributed to patient outcomes?

10. Please describe the process your institution used in supporting your application to MFSP:

11. We would welcome any additional thoughts, comments, or suggestions you might have about improving the Macy Faculty Scholars Program.

Thank you for your time. If you would like to share other thoughts or have any questions, please contact: Larry Gruppen, PhD (lgruppen@umich.edu)
